# Extension of secular perturbation theory for asteroids

**Published:** 2004-04-01

**Authors:** Yoshihide Kozai

**Affiliations:** Gunma Astronomical Observatory 6860-86, Nakayama, Takayama-mura, Agatsuma-gun, Gunma 377-0702

**Keywords:** Secular perturbations, asteroids, stellar three body problem

## Abstract

In this paper the theory of secular perturbations of asteroids with high inclinations and eccentricities (1962) is reviewed and extended analytically for orbits of Kuiper-belt objects and the problem of stellar three bodies consisting of a binary and a distant third body. The theory thus extended seems to be consistent with numerical results published in various papers on subjects like periodic comets, planets of extra-solar systems, star clusters and binaries of black-holes.

## Introduction

In the previous paper1) Kozai showed that when the inclination and/or the eccentricity are large enough the secular perturbation theory for asteroids exhibits orbital element variations quite different from those described in classical textbooks of celestial mechanics. In fact such textbooks assume that inclinations and eccentricities of asteroids are so small that their squares can be neglected. The 1962 paper is republished2) with comments by Marsden3) when the American Astronomical Society celebrated its centennial anniversary.

In the paper1) Kozai shows that $\sqrt{a(1-e^{2})}\;\text{cos }i$, *a*, *e* and *i* being the semi-major axis, eccentricity and inclination, respectively, is constant under the assumption that the disturbing planet moves in a circular orbit. It was also shown that after averaging the disturbing function with respect to the mean anomaly of the asteroid and the mean longitude of the disturbing planet, the equations of motion are reduced to a system of one degree of freedom. Moreover, it was demonstrated that a stationary solution with fixed argument of perihelion as well as constant eccentricity and inclination may exist if the value of (1 − *e*^2^) cos^2^ *i* is smaller than 0.6. Around the stationary solution there is a region, in which the argument of perihelion librates around 90° or 270° and the eccentricity and inclination vary widely with the argument of perihelion in and near libration regions.

Although any difference among various secular perturbation theories cannot be detected clearly by tracing asteroid motions for decades or so, the validity of the theory was verified by integrating numerically equations of motion for a long time.4) Subsequently Kozai5) showed that the method can be applied to the case, for which several disturbing planets move in the same plane with circular orbits and also applied the theory to comets5) and Pluto.6) By using the theory, stability of asteroid motions, particularly with high eccentricity, was discussed4),5) and members of Pallas family with high inclination were identified.5)

As Kuiper-belt objects, near-earth objects and planets of extra-solar systems are discovered, it appears that there are many objects, for which the inclinations and/or eccentricities are large.7),8) Therefore, the theory was applied to try to explain how such high inclinations and eccentricities can be produced by gravitational attraction of a third body.

The theory also provides a mechanism for increasing the eccentricity of a binary system due to a distant third body in a highly inclined plane around the binary. When the eccentricity becomes larger, tidal dissipation effects and the gravitational radiation emitted from the system for the case of a black-hole binary are increased so that the orbital size of the binary is significantly reduced. In order to apply the theory to super-massive black-holes, the additional effect of the general relativity theory must be considered.9)

In this paper the author tries to formulate analytical expressions also for the cases of Kuiper-belt objects, for which the disturbing planets are moving inside their orbits, and for that of the stellar three body problem consisting of a binary and a distant third body.

## Equations of motion

Let us assume that the three bodies, *S* with mass *m*_0_, *P* with mass *m* and *J* with mass *m*′, are moving under their mutual attraction. Then the force function, *U*, is written with the gravitational constant, *k*^2^, as *U* = *k*^2^(*m*_0_*m/R* +*m*_0_*m*′*/R*′ +*mm*′*/*Δ), where *R*, *R*′ and Δ are, respectively, the distances between *S* and *P*, *S* and *J* and *P* and *J*.

When their coordinates in an inertial system are written as **r***_S_*, **r***_P_* and **r***_J_*, the equations of motion for the three bodies are formulated in the usual way. Then the relative coordinates, **r** of *P* with respect to *S* and **r**′ of *J* with respect to the center of gravity of *S* and *P*, are introduced as **r** = **r***_P_* − **r***_S_*, **r**′ = **r***_J_* − (*m*_0_**r***_S_* +*m***r***_P_* )*/*(*m*_0_ +*m*).

When *r/r*′ is smaller than 1, the distances, *R*, *R*′ and Δ′, are expressed in terms of *r*, *r*′ and *s*, with *s*= (**r***,***r**′)*/rr*′, by the following equations in addition to *R* = *r*;

[1] $$\begin{array}{l} R^{2}={r^{\prime }}^{2}\;\left[1+2\frac{m}{m_{0}+m}\frac{r}{r^{\prime }}s+{\left(\frac{m}{m_{0}+m}\frac{r}{r^{\prime }}\right)}^{2}\right],\\ \Delta^{2}={r^{\prime }}^{2}\;\left[1-2\frac{m_{0}}{m_{0}+m}\frac{r}{r^{\prime }}s+{\left(\frac{m_{0}}{m_{0}+m}\frac{r}{r^{\prime }}\right)}^{2}\right]. \end{array}$$

Using the Legendre function, *P_n_*, the force function becomes

[2] $$U=k^{2}\;\left[\frac{m_{0}m}{r}+\frac{(m_{0}+m)m^{\prime }}{r^{\prime }}+\frac{m_{0}mm^{\prime }}{m_{0}+m}\frac{r^{2}}{{r^{\prime }}^{3}}P_{2}(s)\right].$$

Higher order terms with respect to *r/r*′ are neglected. Hence the equations for **r** and **r**′ are,

[3] $$\begin{array}{l} \frac{d^{2}r}{dt^{2}}=\frac{m_{0}+m}{m_{0}m}\frac{\partial U}{\partial r}=k^{2}\frac{\partial }{\partial r}\;\left[\frac{m_{0}+m}{r}+m^{\prime }\frac{r^{2}}{{r^{\prime }}^{3}}P_{2}(s)\right]\\ =k^{2}\frac{\partial }{\partial r}\frac{m_{0}+m}{r}\times \;\left[1+\frac{m^{\prime }}{m_{0}+m}\;{\left(\frac{a}{a^{\prime }}\right)}^{3}{\left(\frac{r}{a}\right)}^{3}{\left(\frac{a^{\prime }}{r^{\prime }}\right)}^{3}P_{2}(s)\right], \end{array}$$

[4] $$\begin{array}{l} \frac{d^{2}r^{\prime }}{dt^{2}}=\frac{m_{0}+m+m^{\prime }}{(m_{0}+m)m^{\prime }}\frac{\partial U}{\partial r^{\prime }}=k^{2}\frac{\partial }{\partial {\text{r}}^{\prime }}\;\left[\frac{m_{0}+m+m^{\prime }}{r^{\prime }}+\frac{(m_{0}+m+m^{\prime })m_{0}m}{{\left(m_{0}+m\right)}^{2}}\frac{r^{2}}{{r^{\prime }}^{3}}P_{2}(s)\right]\\ =k^{2}\frac{\partial }{\partial r^{\prime }}\frac{m_{0}+m+m^{\prime }}{r^{\prime }}\times \;\left[1+\frac{m_{0}m}{{\left(m_{0}+m\right)}^{2}}\;{\left(\frac{a}{a^{\prime }}\right)}^{2}{\left(\frac{r}{a}\right)}^{2}{\left(\frac{a^{\prime }}{r^{\prime }}\right)}^{2}P_{2}(s)\right]. \end{array}$$

The equations [3] and [4] show the main terms for the two body problem and the disturbing terms due to the third body for *P* and *J* and the disturbing terms are used as disturbing functions in the following discussion.

The equations of motion are also written by means of the following canonical variables;

**Table:** 

*L* = *μa*^1^*^/^*^2^,	*l*=mean anomaly*,*
$G=L\sqrt{1-e^{2}}$,	*g*= argument of perihelion*,*
*H* = *G*cos*i*,	*h* = longitude of ascending node*,*

where *μ*^2^ =*k*^2^(*m*_0_+*m*′) and *F* =*k*^2^*/*(2*L*^2^)+disturbing function.

In this paper only the secular perturbations are treated by averaging the disturbing function with respect to the mean anomaly of the disturbed body and the mean longitude of the disturbing body. This makes *L* likewise *a* constant. The problem discussed here is restricted to the case, for which *J* is not perturbed and the potential of the disturbing force is axially symmetric so that *h* does not appear in the disturbing function. It follows that *H* is constant, hence *H/L* is also constant in the system discussed. In this way the system is reduced to that of one degree of freedom with *G* and *g* as the variables. The secular part of the disturbing function is denoted by *R_s_* and the parameter, Θ, is introduced as,

[5] $$\Theta =H/L=\sqrt{1-e^{2}}\;\text{cos }i.$$

Accordingly, the equations of motion to be solved are reduced to; *dG/dt* = *∂R_s_/∂g*, *dg/dt* = −*∂R_s_/∂G*, where *R_s_* is constant since the time does not appear explicitly in *R_s_*. In the following sections the equations of motion are solved for several cases.

## Asteroids

For the case of asteroids moving mostly inside Jupiter’s orbit (*a*=5.20AU, *e*=0*.*049, $i=0_{\cdot }^{\circ }3$ referred to the invariable plane of the solar system), *S*, *J* and *P* are, respectively, the sun, Jupiter, and the asteroid and the mass of the asteroid, *m*, can be assumed to be zero. The averaged part of the disturbing function, *R_s_*, is then written by neglecting terms with the factor (*a/a*′)^4^ and replacing *m*′*/m*_0_ by *m*′;

[6] $$R_{s}=-\frac{1}{16}\frac{\mu^{2}}{a^{\prime }}m^{\prime }\;{\left(\frac{a}{a^{\prime }}\right)}^{2}\;\left[\left(1-3\;{\left(\frac{H}{G}\right)}^{2}\right)\;\left(5-3\;{\left(\frac{G}{L}\right)}^{2}\right)-15\;\left(1-{\left(\frac{H}{G}\right)}^{2}\right)\;\left(1-{\left(\frac{G}{L}\right)}^{2}\right)\;\text{cos }2g\right]\cdot$$

Note that odd-order Legendre terms disappear since Jupiter’s eccentricity is assumed to be zero.

Since it is assumed that Jupiter moves in a circular orbit and the orbital plane of Jupiter is taken as the reference plane, the longitude of the ascending node, *h*, disappears in the disturbing function. Then the equations of motion are reduced to;

[7] $$\frac{dG}{dt}=\frac{\partial R_{s}}{\partial g}=\frac{15}{8}m^{\prime }nL\;{\left(\frac{a}{a^{\prime }}\right)}^{3}\left(1-{\left(\frac{H}{G}\right)}^{2}\right)\;\left(1-\;{\left(\frac{G}{L}\right)}^{2}\right)\;\text{sin }2g,$$

[8] $$\frac{dg}{dt}=-\frac{\partial R_{s}}{\partial G}=\frac{3}{8}m^{\prime }n\;{\left(\frac{a}{a^{\prime }}\right)}^{3}\;\frac{L}{G}\;\left[5\;{\left(\frac{H}{G}\right)}^{2}-{\left(\frac{G}{L}\right)}^{2}-5\;\left(\;{\left(\frac{H}{G}\right)}^{2}-{\left(\frac{G}{L}\right)}^{2}\right)\;\text{cos }2g\right],$$

where *n* is the mean motion and *μ* is replaced by *na*^3^*^/^*^2^.

If 2*g* is 0° or 180°, *dG/dt* vanishes. And if cos 2*g* = 1 it is seen that *dg/dt* does not vanish, whereas if cos2*g* = −1, *dg/dt* can vanish if the equation, 3(*G/L*)^4^ =5Θ^2^, has a root for (*G/L*)^2^ =1− *e*^2^. That is, if Θ^2^ is smaller than 0.6, the equations [7], [8] can have a stationary solution with 2*g* = 180°. When *a/a*′ attains a larger value, this relation is modified. However, generally as *a/a*′ becomes larger, a stationary solution exists even for a little larger value of Θ.

The theory can be extended to the case, for which planets other than Jupiter are included as disturbing bodies under the condition that all are in the same plane with circular orbits. In this paper Saturn (*a* = 9.55AU, *e* = 0*.*056, $i=0_{\cdot }^{\circ }9$), Uranus (*a* = 19.22AU, *e* = 0*.*045, $i=1_{\cdot }^{\circ }0$) and Neptune (*a*=30.11AU, *e*=0*.*009, $i=0_{\cdot }^{\circ }7$) are included. Then with given values of *a* and Θ, numerical values of *R_s_*, which is the sum of the averaged values of *m*′*/*Δ for all the planets, are computed for various sets of 2*g* and $X=G/L=\sqrt{1-e^{2}}$ and equi-*R_s_*-value curves are drawn. When the initial values of 2*g* and *X* are assigned, the motion can be followed along an equi- *R_s_*-value curve.

**Fig. 1:**
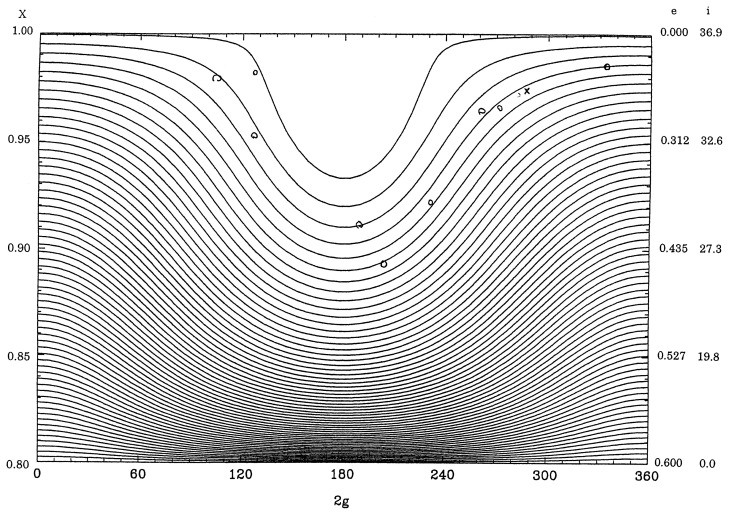
Equi-*R_s_*-value curves for *a* = 2.77AU and Θ=0*.*8. Pallas family asteroids are plotted.

**Fig. 2:**
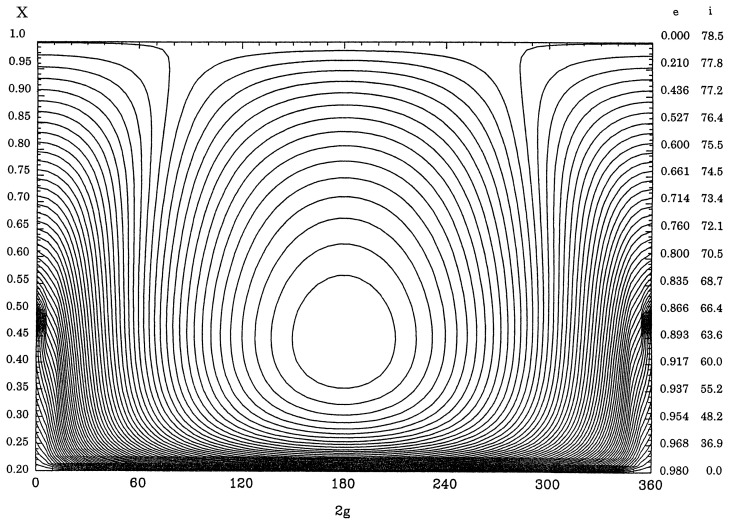
Equi-*R_s_*-value curves for *a* = 2.77AU and Θ=0*.*2.

In Fig. 1 equi-*R_s_*-value curves are shown for *a*= 2.77AU and Θ = 0*.*8 and those for Θ = 0*.*2 with the same value of *a* are displayed in Fig. 2. The horizontal axis represents values of 2*g* between 0° and 360°, whereas the vertical axis displays $X=\sqrt{1-e^{2}}$. The values of *X* are shown along the left vertical axis and along the right axis are shown values of *e* and *i*.

Along the axes of 2*g* = 0° and 360° in the figures, the value of *R_s_* is increased as *X* is decreased, that is, downwards. On the other hand along the line of 2*g* = 180°, the value of *R_s_* is decreased as *X* is decreased from 1 and attains its minimum value at the stationary point and then increases, again, up to *X* = Θ. The motion of *g*, which is proportional to −*∂R_s_/∂G*, or equivalently −*∂R_s_/∂X*, is direct except in the part above the stationary point along the line of 2*g* = 180°. Therefore, the libration region appears around the stationary point and the argument of perihelion librates there. In the other regions the motion of the argument of perihelion is direct.

Figure 1 is for the asteroid (2)Pallas represented by *×* and some of the Pallas family members are denoted by ○.5) Although the eccentricities of the Pallas family asteroids are scattered between 0.0 and 0.3, their original eccentricities should have been nearly equal to each other. Figure 2 shows that the eccentricity of an asteroid along the boundary curve of the libration region can vary between 0.0 and 0.96. In fact more than 10 asteroids are found to be in libration regions in such figures.10)

Outside libration region the eccentricity for any curve reaches its minimum value at 2*g* = 0°, that is, when the major axis of the orbit is in the plane of the disturbing planets, and the maximum at 2*g* = 180° when the major axis is far from the reference plane. In the libration region the major axis never lies in the plane of the disturbing planets and the eccentricity reaches both the maximum and the minimum values at 2*g* = 180°. This means that asteroids, even if their aphelia are outside Jupiter’s orbit, can avoid approaching Jupiter very closely. However, if any equi-*R_s_*-value curve in the lower part of figures is followed, the eccentricities do not change enough to avoid any close approach. This is the case for periodic comets,5) for which usually the value of *R_s_* is large since most comets frequently approach Jupiter.4)

Since effects of the planets other than Jupiter, the nearest planet with the largest mass, are not appreciable, Figs. 1 and 2 are not much different from those, in which only Jupiter is considered. For this case the figures do not depend on the mass of Jupiter, although the motion of *g* is proportional to the mass. Also the figures are not distorted significantly by the value of *a* unless *a/a*′ is very close to 1.

Therefore, it is remarkable to see that the secular perturbations of any high inclination and/or eccentricity asteroid can be roughly estimated by the method discussed here unless it approaches Jupiter very closely. This is verified by comparing the results with those by numerical integrations which take into account of forces by major planets with actual orbits.

## Kuiper-belt objects

Since orbits of Kuiperbelt objects are mostly outside Neptune’s orbit, *r/r*′ is assumed to be larger than 1. Then the disturbing function, *R*, can be expressed as;

[9] $$\begin{array}{l} R=\mu^{2}m^{\prime }{\left(r^{2}+{r^{\prime }}^{2}-2rr^{\prime }s\right)}^{-1/2}\\ =(\mu^{2}m^{\prime }/r)\sum P_{j}(s){\left(r^{\prime }/r\right)}^{j}\cdot \end{array}$$

And for averaging the disturbing function the following relations should be used;

[10] $$\begin{array}{l} \bar{{\left(a/r\right)}^{3}}={\left(1-e^{2}\right)}^{-3/2},\;\;\;\bar{{\left(a/r\right)}^{3}\;\text{cos }2f}=0,\\ \bar{{\left(a/r\right)}^{5}}={\left(1-e^{2}\right)}^{-7/2}\;\left(1+\frac{3}{2}e^{2}\right),\\ \bar{{\left(a/r\right)}^{5}\;\text{cos }2f}=\frac{3}{4}e^{2}{\left(1-e^{2}\right)}^{-7/2},\\ \bar{{\left(a/r\right)}^{5}\;\text{cos}\;4f}=0. \end{array}$$

The second relation shows that cos2*g* term does not appear in *P*_2_. Therefore, in the following expression terms up to *P*_4_ are included, which gives;

[11] $$\begin{array}{ll} R_{s}=\; & -\mu^{2}m^{\prime }\frac{{a^{\prime }}^{2}}{a^{3}}\;[{\left(\frac{L}{G}\right)}^{3}\;\left(-1+3\;{\left(\frac{H}{G}\right)}^{2}\right)/8\\ & \{{\left(\frac{a^{\prime }}{a}\right)}^{2}\;{\left(\frac{L}{G}\right)}^{5}\;[(9/1024)(3-30\;{\left(\frac{H}{G}\right)}^{2}\\ & +35\;{\left(\frac{H}{G}\right)}^{4})\;\left(5-3\;{\left(\frac{G}{L}\right)}^{2}\right)]\\ & -(315/512)\;\left(1-{\left(\frac{H}{G}\right)}^{2}\right)\;\left(1-7\;{\left(\frac{H}{G}\right)}^{2}\right)\\ & \times \left(1-{\left(\frac{G}{L}\right)}^{2}\right)\;\text{cos}\;2g\}]\cdot \end{array}$$

Note that the expression in the first line is the same as that for an artificial satellite around the oblate earth except for the factor depending on *a* and *a*′.

Then the equations of motion are;

[12] $$\frac{dG}{dt}=\frac{\partial R_{s}}{\partial g}=\frac{315}{256}m^{\prime }nL\;{\left(\frac{a^{\prime }}{a}\right)}^{4}\;{\left(\frac{L}{G}\right)}^{5}\;\left(1-{\left(\frac{H}{G}\right)}^{2}\right)\times \left(1-7\;{\left(\frac{H}{G}\right)}^{2}\right)\;\left(1-{\left(\frac{G}{L}\right)}^{2}\right)\;\text{sin }2g,$$

[13] $$\begin{array}{ll} \frac{dg}{dt}=\; & -\frac{\partial R_{s}}{\partial G}=m^{\prime }\;{\left(\frac{a}{a^{\prime }}\right)}^{2}n\;[-\frac{3}{8}{\left(\frac{L}{G}\right)}^{4}\;\left(1-5\;{\left(\frac{H}{G}\right)}^{2}\right)\\ & +[{\left(\frac{a^{\prime }}{a}\right)}^{2}\;{\left(\frac{L}{G}\right)}^{6}\;[\frac{9}{1024}\{-5\;(15-210\;{\left(\frac{H}{G}\right)}^{2}\\ & +315\;{\left(\frac{H}{G}\right)}^{4})+3\;\;{\left(\frac{G}{L}\right)}^{2}\;(9-150\;{\left(\frac{H}{G}\right)}^{2}\\ & +245\;{\left(\frac{H}{G}\right)}^{4})\}-\frac{315}{512}\;\{\left(5-56\;{\left(\frac{H}{G}\right)}^{2}+63\;{\left(\frac{H}{G}\right)}^{4}\right)\;\\ & -3\;{\left(\frac{G}{L}\right)}^{2}\left(3-40\;{\left(\frac{H}{G}\right)}^{2}+49\;{\left(\frac{H}{G}\right)}^{4}\right)\}\;\text{cos }2g]]]\cdot \end{array}$$

The equation for *dG/dt* shows that the amplitude of cos 2*g* term is proportional to (*a*′*/a*)^2^(*L/G*) since there is no sin2*g* term due to *P*_2_. Note that factors such as (*a*′*/a*)^2^ do not appear in cos2*g* term for the asteroid case. The first term in *dg/dt* vanishes if $\Theta =\sqrt{1/5}\;(i=63_{\cdot }^{\circ }4)$, which is the critical inclination of the artificial satellite problem. The amplitude of cos 2*g* term in [13] is usually small unless the eccentricity is very large, so that no stationary solution exists for any moderate value of Θ, whereas a stationary solution may exist for $i=63_{\cdot }^{\circ }4$.

**Fig. 3:**
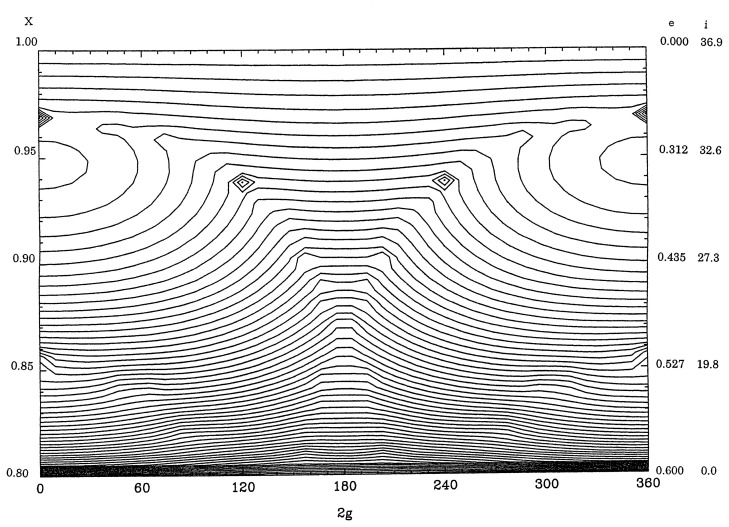
Equi-*R_s_*-value curves for *a* = 40AU and Θ=0*.*8.

**Fig. 4:**
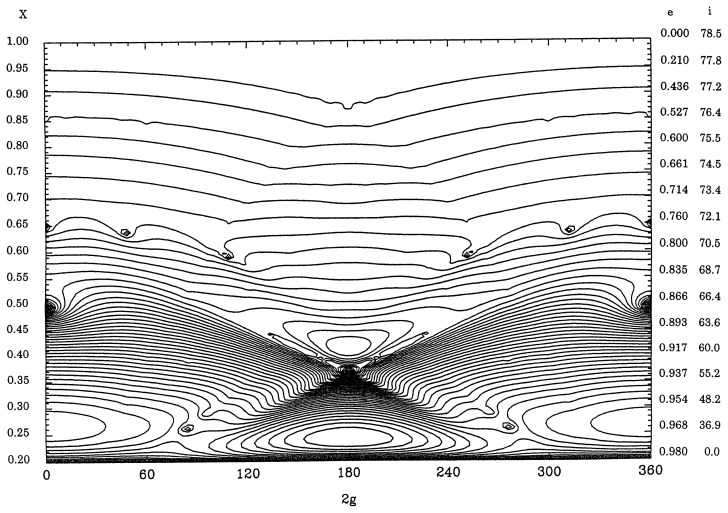
Equi-*R_s_*-value curve for *a* = 40AU and Θ=0*.*2.

Figure 3 displays equi-*R_s_*-value curves for *a* = 40AU and Θ = 0*.*8 and Fig. 4 is for Θ = 0*.*2 with the same value of *a* by including the effect of major planets moving in the same plane with circular orbits. The features are quite different from those of Figs. 1 and 2 since the amplitudes of the variation of *X* as functions of 2*g* are smaller. Some libration regions appear around stationary points on the line of 2*g* = 0°, as these points correspond to local maxima of *R_s_*. However, as there is a high possibility that Kuiper-belt objects approach Neptune closely at 2*g* = 0 if the eccentricity is larger than 0.25, the computation near the vertical axes for these figures are not so accurate.

In Fig. 4 there are two libration regions around points on the line of 2*g* = 180°. The upper libration region is due to the critical inclination ($63_{\cdot }^{\circ }4$) and the lower one represents a stable libration region7) with large eccentricities and moderate values of inclinations. Such libration regions appear also when equi-*R_s_*-value curves are drawn for some of long-period comets like 1P/Halley.5)

**Fig. 5:**
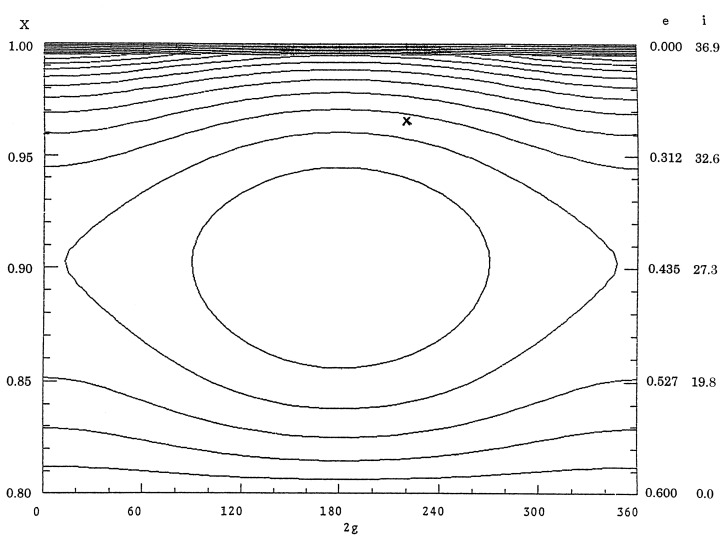
Equi-*R_s_*-value curves for Pluto marked as x, for which *a* = 40AU and Θ=0*.*8.

Figure 5 is for Pluto (*a* = 39.5AU, *e* = 0*.*249, $i=15_{\cdot }^{\circ }6,g=113_{\cdot }^{\circ }8$), for which the critical argument, 3*λ*′ −2*λ* + *ϖ*′, librates around 180°. Here *λ* and *λ*′ are the mean longitudes of Neptune and of Pluto, respectively and *ϖ* is the longitude of the perihelion of Pluto. In fact Pluto is in 2 : 3 mean motion resonance with Neptune.

The computation is made under the assumption that the critical argument is fixed at 180°, that is, the opposition of Neptune and Pluto takes place only at the aphelion of Pluto. Then as the eccentricity becomes larger, the mutual distance at the opposition becomes larger so that the value of *R_s_* becomes smaller. However, when the eccentricity becomes much larger, a close approach to Uranus becomes possible so that the value of *R_s_* becomes larger. Therefore, a shallow libration region appears in Fig. 5 and Pluto, marked as *×*, is near the boundary of the libration region. Similar figures can be drawn for *a* = 48AU of 1 : 2 mean motion resonance case if the critical argument librates around 180°.

If *a*′*/a* attains a smaller value, equi-*R_s_*-value curves for not so large value of the eccentricity becomes nearly horizontal. Therefore, solutions of nearly constant and low eccentricity can exist even though the inclinations are high. However, it is not possible that a high inclination orbit is produced from a low inclination one by this mechanism.

## Stellar three body problem

The increase of the eccentricities of binary orbits in star clusters has frequently been noticed by Valtonen,11) Aarseth12) and others in their numerical integrations of the N-body problem when a third body is in bound motion around a binary. Since many planets of extra-solar systems13),14) and even cool Algol stars15) have eccentric orbits, the importance of the third body is emphasized and the secular perturbation theory provides a mechanism for increasing the eccentricity from nearly zero to large values. By this mechanism the increase of the tidal friction effect and the gravitational wave radiation from black-hole binaries9),16) follows. For the case of a black-hole binary the effect of general relativity should be included.9)

Now *S* with mass, *m*_0_, and *P* with mass, *m*, constitute a binary or a system of a star and its planet and *J* is the third body moving in a circular orbit with the mass, *m*′. The orbit of *J* is also perturbed since the central body is not a point source. Since the motion of *J* is dynamically similar to that for Kuiper-belt objects, *J* can have an orbit of very low eccentricity and high inclination at least during the period when the orbit of the central system is circular. Moreover, if the inclination is nearly 90°, the node, that is, the orbital plane of *J*, does not move much like that of a polar satellite. However, the central orbit is expected to become eccentric afterwards.

To understand how the orbital variations behave as functions of the arguments of peri-astron, dynamical time-scales of *J* and *P* are compared. The disturbing factors for *J* with primed orbital elements and *P* are, respectively, [*mm*_0_*/*(*m*+*m*_0_)^2^](*a/a*′)^2^ and [*m*′*/*(*m*+*m*_0_)](*a/a*′)^3^ according to the equations [3] and [4]. The time-scales are derived by multiplying them by their respective mean motions, *n*′= *k*(*m*_0_+*m*+*m*′)^1^*^/^*^2^*a*′^−3^*^/^*^2^ and *n*=*k*(*m*_0_+*m*)^1^*^/^*^2^*a*^−3^*^/^*^2^. Then the ratio of the time-scale of *P* with respect to *J*, *γ*, is expressed as,

[14] $$\gamma =\sqrt{\frac{a^{\prime }}{a}}\sqrt{\frac{m_{0}+m}{m_{0}+m+m^{\prime }}}\frac{m^{\prime }(m_{0}+m)}{m_{0}m}.$$

For the problem treated here *a*′*/a* is larger than 1. If *m*′is much larger than the other masses, *γ* attains a very large value, and therefore, the variations of orbital elements of *P* are much more rapid than those of *J*. This corresponds to the lunar problem and if the orbit of *J*, the sun, is assumed to be circular, the orbit of *P*, the moon, can be traced by the theory treated here. On the other hand if *P* is a planet with a small value of *m* moving around *S*, the orbits of *S* and *J* are nearly those of the two body problem, and, therefore, the configuration of the system is very similar to that of the case for asteroids. Also if *a*′*/a* attains a very large value, the orbit of *J* with respect to the center of mass of *S* and *P* can be also approximated by that of the two body problem.

And if *γ* attains a large value with a set of the three masses and *a*′*/a*, the orbital variations for *J* are slower. Moreover, the amplitude of the cos 2*g*′ term for *J* is small because of the factor, (*a/a*′)^2^. This concludes that the orbital variations for *P* take place more rapidly and more widely than those for *J* under the assumptions stated above. In fact if *γ* attains a large value, *J* can move in a circular orbit and a fixed plane. Also if *a*′*/a* is sufficiently large and if the outer inclination is almost 90°, the node, that is the orbital plane, does not move much.

Therefore, as far as the orbit of *J* can be assumed to be circular in a fixed plane, the secular perturbation theory discussed here can be applied to the stellar three body problem. In fact direct three-body integrations show that the restriction to large mass for the third body is not necessary17) and, in principle, any value can produce the desired effect except that the time-scale may be long.18),19)

In summary, in this paper it is shown that Figs. 1 and 2 represent dynamical characteristics of the motion of any system with potential similar to the case of asteroids discussed previously. For such a system once the value of $\Theta =\sqrt{1-e^{2}}\;\text{cos }i$ is given the motion can be described. Thus for this case both the inclination and the eccentricity can vary widely as functions of twice the argument of perihelion if the value of Θ is sufficiently small. By this way the actual motion of such an asteroid can be estimated.

For orbits of Kuiper-belt objects, Figs. 3 and 4 show that the inclinations and the eccentricities are not increased appreciably from zero according to the secular perturbation theory unless there are mean-motion and/or secular motion resonance. Still low eccentricity and high inclination orbits can exist for a long time interval.

For the stellar three body problem treated in this paper the distant third body can increase the eccentricity of the orbits of the binary stars or a planet around a component of double stars, when the orbital plane of the third body is highly inclined to that of the binary or the system of a star and a planet under some conditions. In fact there are such cases among planets in extra-solar system, triple stars in star clusters and others as simulation computations show.

Periodic comets usually follow equi-*R_s_*-value curves appearing in lower part of Fig. 2, for example, as their inclinations take moderate values whereas the eccentricities are large and do not change much with the argument of perihelion. It is also possible that some of comets like 1P/Halley follow curves similar to those in the lower libration region in Fig. 4 as their orbits are extended even over Neptune.

In conclusion numerical results published in several papers on asteroids, Kuiper-belt objects, comets, planets of extra-solar systems, star clusters, stellar three body problem as well as other work seem to support the extended theory presented here.
